# In vitro study on the disinfectability of two split-septum needle-free connection devices using different disinfection procedures

**DOI:** 10.3205/dgkh000260

**Published:** 2015-12-09

**Authors:** Steffen Engelhart, Martin Exner, Arne Simon

**Affiliations:** 1 Institute for Hygiene and Public Health, Universitätsklinikum Bonn, Germany; 2 Pediatric Oncology and Hematology, Children's University Hospital, Homburg, Saar, Germany

**Keywords:** needle-free connection devices (NFC), central venous catheter, hub disinfection, octenidine/propanol, 2-propanol

## Abstract

This in vitro study investigated the external disinfection of two needle-free connection devices (NFC) using Octeniderm^®^ (spraying and wiping technique) vs. Descoderm^®^ pads (wiping technique). The split-septum membrane of the NFC was contaminated with >10^5^ CFU *K. pneumoniae or S. epidermidis*. The efficacy of the disinfection at 30 sec. exposure time was controlled by taking a swab sample and by flushing the NFC with sterile 0.9% sodium chloride solution. Disinfection with octenidine dihydrochloride 0.1 g, 1-Propanol 30.0 g, and 2-Propanol 45.0 g in 100 g solution was highly effective (CFU reduction ≥4 log) against both microorganisms, whereas the use of 63.1 g 2-Propanol in 100 ml solution led to residual contamination with *S. epidermidis*. Our investigation underlines that (i) in clinical practice disinfection of NFCs before use is mandatory, and that (ii) details of disinfection technique are of utmost importance regarding their efficacy. Our investigation revealed no significant differences between both split-septum NFC types. Clinical studies are needed to confirm a possible superiority of disinfectants with long-lasting residual antimicrobial activity.

## Zusammenfassung

In einer Reihe von in vitro-Versuchen wurde die Desinfizierbarkeit von zwei nadelfreien Split-Septum-Konnektionsventilen (NFC) mit einem Hautantiseptikum auf Basis von Octenidindihydrochlorid 0,1 g, 1-Propanol 30,0 g und 2-Propanol 45,0 g in 100 g Lösung mittels Sprüh- und Wischdesinfektion mit steriler Kompresse vs. Wischdesinfektion mit vorgetränkten Läppchen mit 63,1 g 2-Propanol in 100 ml Lösung untersucht. Dabei wurde die äußere Membran des NFC mit >10^5^ KbE *K. pneumoniae oder S. epidermidis* kontaminiert. Die Überprüfung des Desinfektionserfolgs bei einer Einwirkungszeit von 30 Sek. erfolgte mittels Abstrich und Durchspülmethode.

Die Desinfektion mit dem Hautantiseptikum erwies sich in Bezug auf beide Bakterienspezies als hocheffektiv. Hingegen konnte nach der hier durchgeführten Methode der Desinfektion mit vorgetränkten Läppchen in einigen Versuchsreihen mit *S. epidermidis* keine ausreichende Dekontamination beider NFC-Typen erreicht werden.

Die vorliegende Untersuchung unterstreicht, dass NFCs im klinischen Einsatz vor einer geplanten Konnektion immer der Desinfektion bedürfen und dass die Technik der Desinfektion von entscheidender Bedeutung ist. Eine klare Überlegenheit eines der beiden Konnektoren konnte nicht festgestellt werden. Ob Desinfektionsmittel mit einer Remanenzwirkung tatsächlich überlegen sind, muss in weiterführenden Studien untersucht werden.

## Introduction

Besides full barrier precautions at insertion of central venous catheters (CVCs) and removal of unnecessary catheters as soon as possible [1], [2], maintenance care plays a major role in prevention of catheter bloodstream infections (BSI) originating from CVC surfaces or access ports [3]. 

There is a broad consensus that before any manipulation the catheter hub or other venous access sites have to be thoroughly disinfected [3], [4], [5], [6], [7], [8]. If frequent manipulations are necessary, a needle-free connection device (NFC) can be advantageous [9], as its disinfection is easier than disinfection of a three-way stopcock [10], [11], [12]. 

The manufacturers of NFCs are obliged to supply the user with information as to which disinfection methods and preparations are applicable for decontamination of the particular NFC-model’s access site during clinical use. Some reports indicate that NFCs can even raise the risk of catheter-related bloodstream infections [13], in particular positive pressure NFCs [14], [15], [16], [17], and in case of insufficient education and training concerning the correct use and disinfection of these devices [18], [19], [20].

In order to reach a high compliance (>95%) with the correct use of NFCs [21], [22], the disinfection method must be practicable. A good example of a non-practical procedure is cited by Adams et al. [23]: 

„…firmly applying individual swabs containing 70% (v/v) isopropyl alcohol (IPA) (Sterets; Seton Healthcare, Oldham,UK) to the compression seal and rotating five times through 360°. The 70% (v/v) IPA was subsequently allowed to dry for 2 min.”

The time needed to complete the whole disinfection procedure must not exceed 30 seconds which can reasonably be used for additional hand disinfection. 

Some experts recommend the use of a disinfectant combination including a long-lasting residual disinfectant activity, e.g. alcohol with chlorhexidine or octenidine, for hub disinfection [24], [25], [26] in analogy to skin antisepsis with chlorhexidine 2% / isopropanol 70% during insertion of CVCs [3]. 

This in vitro study investigates the disinfection of two split-septum NFC models (BD Q-Syte^®^ und MicroClave^®^) using (1) an alcohol-based ready to use tissue (Descoderm^®^ Pads) or (2) a propanol-octenidine containing disinfectant spray (Octeniderm^®^) under controlled conditions.

## Materials and methods

### NFC types

We investigated two different split-septum needle-free connection devices (NFC): BD Q-Syte^®^ (Becton Dickinson, Heidelberg, Deutschland) [23], [10], and MicroCLAVE (NeoCare GmbH, Lüdenscheid, Deutschland) [27], [28], [29].

### Test microorganisms and inoculation of the NFC membrane

The test microorganisms were *Staphylococcus epidermidis* (ATCC 12228) and *Klebsiella pneumoniae* subspecies pneumoniae (ATCC 13882). The test microorganisms were calibrated at a concentration of 10^8^ CFU/mL, after dilution (1:10 with 0.9% sodium chloride solution) an aliquot of 10 µl was inoculated on the NFC membrane (inoculum >10^5^ CFU/NFC membrane) by pipette. Under the laminar air flow bench, the inoculum was dried over a period of 30 min. 

### Disinfectants 

Descoderm Pads^®^ (Dr. Schumacher GmbH, Melsungen, Germany) are single packaged pre-moistured pads, size: 6×3×8 cm, containing 63.1 g 2-Propanol in 100 ml solution as active component. The pads are commissioned for skin antisepsis before injection or puncture (exposure time 15 seconds). According to the manufacturer, the pads can also be used to disinfect alcohol-resistant surfaces of medical devices (e.g., the rubber surface of infusion bottles) [30].

Octeniderm^®^ (Schülke & Mayr GmbH, Norderstedt, Germany) was used as spray, containing octenidine dihydrochloride 0.1 g, 1-propanol 30.0 g and 2-propanol 45.0 g in 100 g solution as active component. According to the manufacturer, the spray is commissioned for skin antisepsis before surgical operations as well as catheterization or puncture of blood vessels. 

Both antiseptics are listed by the German Disinfectants Commission in the Association for Applied Hygiene.

### Disinfection procedures

**Descoderm^®^ pad:** The NFCs were placed on sterile gauze, and the connector was disinfected by wiping the connecting surface of the device with the pad one time clockwise with moderate digital pressure ensuring to reach the complete surface. The exposure time of the disinfectant was 30 seconds (maximum, 40 seconds). 

**Octeniderm^®^:** The NFCs were placed on sterile gauze, and the connecting surface of the device was disinfected with 4 puffs of the Octeniderm^®^ sprayer. Then, the surface of the NFC was wiped with the Octeniderm^®^ moistened sterile gauze with moderate digital pressure one time clockwise. After that, another two puffs of the Octeniderm^®^ spray were applied. Corresponding to the Descoderm^®^ pad procedure, the exposure time of the disinfectant was at least 30 seconds (maximum, 40 seconds). The doubled positive controls remained without disinfection.

### Laboratory analysis

**Contact samples of the connector membrane:** The outer surface of the membrane including the split was swabbed with a sterile 0.9% NaCl moistened swab (Greiner, Germany; article number 420180); the sample was directly transferred to Columbia 5% sheep blood (SB) and cultured for 24 h at 36°C. For the positive control the outer surface of the membrane including the split was swabbed with a sterile 0.9% NaCl moistened swab; the tip of the swab was vortexed in a tube containing 4.5 ml 0.9% NaCl and processed for quantitative culture using standard dilution techniques (Columbia 5% SB; 24 h / 36°C)

**Flushing samples:** A sample of 100 ml sterile NaCl 0.9% saline flush for each device was collected in a separate sterile membrane filter funnel unit; the membrane filter was then transferred to the surface of a Columbia 5% plate (incubated for 24 h / 36°C). For the positive control a sample of 100 ml sterile NaCl 0.9% saline flush for each device was collected in a separate sterile glass and processed for quantitative culture using standard dilution techniques (Columbia 5% SB; 24 h / 36°C). 

For any combination of the two connectors, disinfection procedures, sample types, and test microorganisms, a total of 8 separate samples and corresponding 2 positive controls each were conducted. Reduction factors were calculated as the differences between the single results from the arithmetic means (log) and the corresponding positive controls (log), respectively. 

## Results

Table 1 (Tab. 1) presents the results of the contact (swab) samples, Table 2 (Tab. 2) those of the flushing samples. Regarding both sample types, we found good recovery (nearly throughout <1 log difference compared with the primary inoculum) in the doubled control samples.

After disinfection with Octeniderm^®^ both NFC types revealed no residual contamination in all contact and flushing samples (both test microorganisms with >4 log reduction factor). 

In contrast, disinfection with Descoderm^®^ pads yielded discordant results: contamination with *K. pneumonia* was completely removed from both NFC types (>4 log reduction factor), but contamination with *S. epidermidis* was not completely eliminated. 6 of 8 contact samples from BD Q-Syte^®^ as well as 2 of 8 flushing samples yielded <3 log reduction factor. The corresponding results for MicroCLAVE^®^ were 1 of 8 contact samples and 4 of 8 flushing samples (resulting reduction factors only between 0.5 to 2.5 log). 

## Discussion

Both split septum NFC models could successfully be disinfected using the Octeniderm^®^ procedure as described above within 30 seconds. Even with a high artificial inoculum (>10^5^ CFU), creating a worst-case scenario compared to bacterial contamination of NFCs in clinical practice [31], no bacterial pathogens (*Klebsiella pneumoniae* or *S. epidermidis*) could be detected on the membrane or in the infusate after disinfection. The exposure time of 30 seconds can favorably be used to conduct hand disinfection before the NFC is accessed. 

In contrast, the Descoderm^®^ pad procedure did not show sufficient efficacy regarding inactivation of *S. epidermidis* in several samples (<4 log reduction and/or detection of microorganisms in flushing samples). *S. epidermidis* is one of the most frequently detected Gram-positive microorganisms detected on hands and hand contact surfaces/fomites and one of the leading pathogens in catheter-related bloodstream infections [3]. 

Apparently, our results are in contradiction with results from other authors on the efficacy of Descoderm^®^ pads [30], [10]. In addition, the manufacturer of the BD Q-Site^®^ NFC recommends wiping the membrane of the NFC for 60 seconds with a moistured pad containing propanol as active component (e.g. Descoderm^®^; pers. communication with Becton Dickinson, Oct. 07/2015). For clarification of this discrepancy, the disinfection procedure has to be discussed in detail. Trautmann et al. describe a disinfection procedure of a different NFC model (Bionecteur^®^, Vygon GmbH, Aachen) as “vigorous circular wiping disinfection of the membrane” and subsequent waiting (30 seconds) until the membrane has fully dried [30]. Thus, our disinfection procedure implies a less intensive mechanic component (just a single wiping course at moderate pressure), on the other hand a shorter exposure time (30 sec vs. 60 sec) compared to the manufacturer’s recommendation.

The different outcomes of the Descoderm^®^ pad resp. the Octeniderm^®^ procedure must also be discussed: several authors described the long-lasting residual disinfectant activity of octenidine in vitro [32] and in vivo when applied on the skin and mucous membranes [33], [34], [35]. However, it remains to be elucidated whether this effect translates into superior clinical efficacy in terms of BSI prevention. At least, some non-randomized clinical cohort studies [24], [25], [26], including a study on a bundle of preventive interventions from a German pediatric oncology department [36] seem to argue for a substantial benefit. It can also not fully be ruled out that octenidine residues could have had an inhibitory effect on the culturability of regained microorganisms in vitro, however, in the case of the flushing samples this seems improbable due to the high dilution factor and no inhibition zone of the flushing sample in a disc diffusion assay. Also, the different tissue characteristics of the pads versus the gauze could have played a minor role (e.g., higher surface adhesion of the gauze). 

In summary, we conclude that (i) the clinical use of NFCs mandatorily requires a previous disinfection before any connection, and (ii) the detailed technique of the disinfection procedure is of utmost importance and thus should be thoroughly defined, educated and trained.

We found no clear superiority of one of the NFC models examined in our study, at best a lower rate of positive flushing samples in case of the BD Q-Syte^®^. Further studies are needed regarding a possible superiority of disinfectant combinations revealing a long-lasting residual disinfectant activity in this clinical context.

## Notes

### Competing interests

The study was part of a project “Intervention bundles on prevention of catheter-related bloodstream infections in pediatric Oncology”, which was funded by the companies Schülke & Mayr GmbH, Norderstedt, Germany, and Becton Dickinson, Heidelberg, Germany. 

In advance to the study, an open discussion concerning the scientific (experimental) state-of-the-art took place including experts from both companies. None of the companies had any influence on study design, realization and evaluation of the study, or on this publication.

## Figures and Tables

**Table 1:**
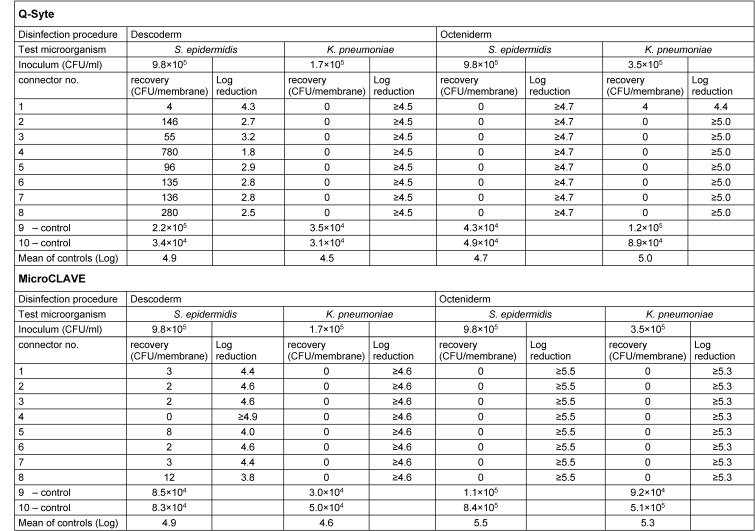
Results of contact samples of the connector membrane

**Table 2:**
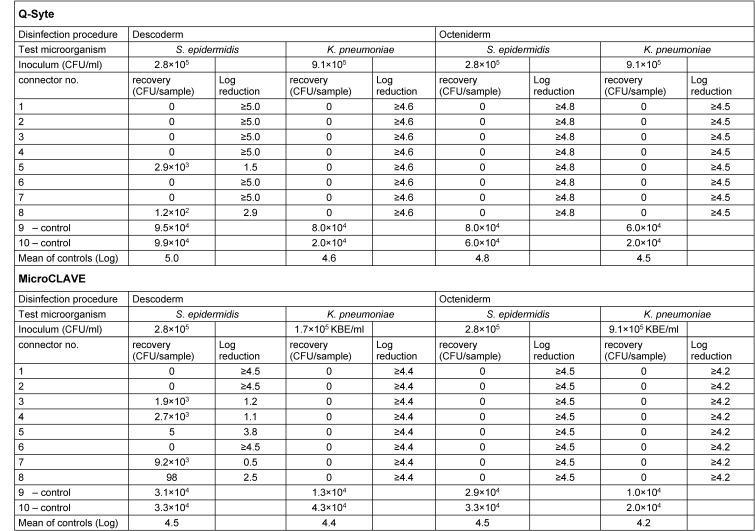
Results of flushing samples
